# Chromosome level assembly and annotation of *Cuscuta campestris* Yunck. (“field dodder”), a model parasitic plant

**DOI:** 10.1093/g3journal/jkaf193

**Published:** 2025-08-20

**Authors:** Juan D Cerda-Herrera, Huiting Zhang, Eric K Wafula, Supral Adhikari, So-Yon Park, Sarah B Carey, Alex Harkess, Paula E Ralph, James H Westwood, Michael J Axtell, Claude W dePamphilis

**Affiliations:** Bioinformatics and Genomics Ph.D. Program, Huck Institutes of the Life Sciences, The Pennsylvania State University, University Park, PA 16802, United States; Department of Horticulture, Washington State University, Pullman, WA 99164, United States; Department of Biology, The Pennsylvania State University, University Park, PA 16801, United States; Division of Plant Science and Technology, University of Missouri, Columbia, MO 65211, United States; Division of Plant Science and Technology, University of Missouri, Columbia, MO 65211, United States; HudsonAlpha Institute for Biotechnology, Huntsville, AL 35806, United States; HudsonAlpha Institute for Biotechnology, Huntsville, AL 35806, United States; Department of Biology, The Pennsylvania State University, University Park, PA 16801, United States; School of Plant and Environmental Sciences, Virginia Tech, Blacksburg, VA 24061, United States; Department of Biology, The Pennsylvania State University, University Park, PA 16801, United States; Department of Biology, The Pennsylvania State University, University Park, PA 16801, United States

**Keywords:** *Cuscuta campestris*, reference genome, parasitic plants, genome sequence, genome browser, comparative genomics, plant genomics, genome annotation, polyploidy, mobile molecules, RUBY transformation, genome assembly

## Abstract

We present the first chromosome-level genome assembly and annotation for the genus *Cuscuta*, a twining and leafless parasitic plant of the morning glory family (Convolvulaceae). *C. campestris*, the study species, is a widely studied model parasite, due in part to its worldwide occurrence as a weed of agricultural and natural plant communities. The species has served as a model parasite for studies of parasite biology, haustorium development, growth responses to chemical and light stimuli, gene content and expression, horizontal gene transfer, and interspecies RNA movement and has a recently developed transformation system. The 505 Mb (1C) genome is assembled into 31 chromosomes and supports annotation of 47,199 protein-coding genes, 214 small RNA loci (including 146 haustoria-specific miRNAs), and 3,238 interspecies mobile mRNA loci. *C. campestris* is a recent tetraploid with a high retention of duplicated genes and chromosomes, with less than 8% nucleotide divergence between homoeologous chromosomes. We also show that transformation of *C. campestris* with the RUBY marker system allows visualization of transformed *Cuscuta*-derived fluorescent mobile molecules that have entered the host stem. This genome, with an associated genome browser and BLAST server, will be of value for scientists performing fundamental research in a wide range of molecular, developmental, population, and evolutionary biology, as well as serve as a research tool for studying interspecies mobile molecules, generating genetic markers for species and genotype identification, and developing highly specific herbicides.

## Introduction

**Fig. 1.**
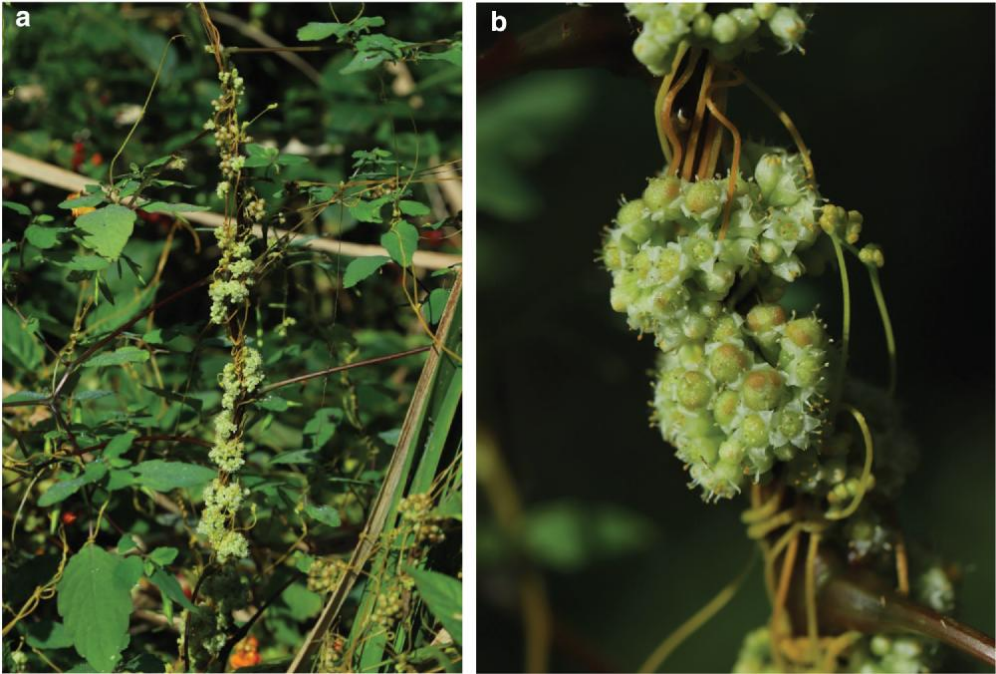
Images of *C. campestris* in habitat. a) *C. campestris* growing on *Impatiens* sp. in Central Pennsylvania, United States. b) Close-up of the plant showing flowers and fruits around its host. Photos by Juan D. Cerda-Herrera.

Parasitic plants form haustoria, feeding structures that attach to host vascular tissues above (stem) or below (root) ground and extract water, minerals, carbohydrates, and other nutrients (Westwood et al. 2010; Nickrent 2020). The genus *Cuscuta* (Convolvulaceae), dodders, with around 200 species, are generally nongreen, twining stem parasites with reduced scale-like leaves, little or no photosynthetic capacity (Schneider et al. 2025), and are dependent upon their hosts for water, minerals, and carbon (Fig. 1) (Van der Kooij et al. 2000). The plastid genomes of *Cuscuta* species have been widely studied, illustrating extreme genome reduction, gene loss, and (in most species) strong functional constraint of core photosynthetic machinery (Funk et al. 2007; McNeal et al. 2007; Banerjee and Stefanović, 2023; Pan et al. 2023). *Cuscuta* are cytogenetically and genomically diverse, with an exceptionally wide range of genome size (0.27 Gb/1C to 34.7 Gb/1C), and with some species bearing holocentric chromosomes (Ibiapino et al. 2022).

*C. campestris* Yunck., field dodder, is the most widely studied *Cuscuta* species due in part to it being a destructive pathogen of agricultural crops (Cooke and Black 1987; Dawson et al. 1994). Native to North America, *C. campestris* is a host generalist able to parasitize a wide range of wild and agricultural dicotyledonous plants, and has spread throughout the world, feeding on diverse crops including tomato and pepper, legumes, and many others (Costea and Tardif 2006). Its invasive capacity is not limited to agricultural ecosystems, as illustrated by its successful introduction to the Galapagos Islands (Costea et al. 2015) and rapid invasive spread in western Kenya (Masanga et al. 2022).

*C. campestris* has become a model species for studies of parasite biology and is a focal species for genomic and transcriptomic studies in parasitic plants. There are 2 published *C. campestris* nuclear genomes (Vogel et al. 2018 and Shahid et al. 2018, a short-read draft), and various transcriptome analyses that have identified differentially expressed genes and potential targets for gene silencing (Ranjan et al. 2014; Bawin et al. 2022). These resources have enabled the documentation of extensive gene loss and the history of polyploidy (Vogel et al. 2018), and new phenomena including interspecies movement of mRNA (Kim et al. 2014; Westwood and Kim 2017) and microRNA (miRNA) (Shahid et al. 2018; Johnson and Axtell 2019), as well as extensive functional horizontal gene transfer (fHGT) (Vogel et al. 2018; Yang et al. 2019), all with implications for parasite–host interactions. Recently developed transformation systems in *C. campestris* (Adhikari et al. 2024; Alles et al. 2025) promise to open many opportunities for functional studies previously unavailable in parasitic plants.

Here, we present a chromosome-level genome assembly and annotation for *C. campestris*, the first such resource in the genus *Cuscuta*. The 505 Mb/1C genome supports an extensive annotation of protein-coding and small RNA genes and is accessible via a public genome server along with supporting transcriptome evidence and alternative annotations. The availability of a high-quality reference genome for *Cuscuta* will facilitate comparative genomic analyses and support an increasingly wide range of genome-enabled experimental research.

## Research methods

### Plant materials

*C. campestris* Yunck. (Costea and Tardif 2006) was grown on tomato, *Solanum lycopersicum* cv. Rutgers, in a greenhouse (Blacksburg, Virginia). The inbred *Cuscuta* strain known informally as “Doddi” had been initially identified as *C. pentagona* in early molecular studies (Kim et al. 2014; Ranjan et al. 2014), but was subsequently identified via phylogenetic analysis as *C. campestris* Yunck. (Shahid et al. 2018; Vogel et al. 2018; Johnson et al. 2019; Yang et al. 2019). The seeds were originally collected from a tomato field near Davis, California, by Dr. Tom Lanini and shared with J.H.W. Doddi was self-fertilized for 4 generations by the J.H.W. Lab before being used to produce tissue for the current work.

### DNA isolation, libraries, and sequencing

Approximately 1 g of frozen stem tip tissue was used for high molecular weight DNA isolations using the NucleoBond HMW DNA kit (Machery-Nagel). To evaluate DNA integrity, 400 ng of DNA was run on a pulsed-field electrophoresis (CHEF) gel for 11 h on a 1% agarose gel, with 1 to 6 s switch times, at 14°C, in 0.5× Tris-borate buffer. The bulk of the DNA formed a band around 140 kb. A PacBio HiFi library was constructed and sequenced at HudsonAlpha Institute for Biotechnology (Huntsville, Alabama) using the SMRTbell Template Prep Kit 1.0, followed by tight sizing on a SAGE ELF instrument. Long-read sequencing was performed using Circular Consensus Sequencing (CCS) mode on a PacBio Sequel II instrument on 4 SMRT cells with a 30-h movie time, 2-h pre-extension, and sequencing chemistry V2.0. The resulting raw data were processed using the CCS4 algorithm. Around 3.4 million reads with an average length of 19.1 kbp were sequenced totaling approximately 66 Gb for an approximately 127× coverage. A Hi-C chromatin capture proximity ligation library was generated at Penn State University using the Proximo Hi-C (Plant) Kit following the recommended protocol and sequenced at the HudsonAlpha Institute for Biotechnology. Approximately 421 million reads of Illumina Hi-C data were produced, resulting in approximately 64 Gb of sequence with an average read length of 144.8 bp, giving 117.3× coverage.

### Genome assembly and evaluation

Genome assembly was done using Hifiasm v0.16.1 with default settings (Cheng et al. 2021). Proximity ligation (Hi-C) sequence data were mapped following the Arima-HiC Mapping Pipeline v02 (Arima Genomics 2019). In short, the pipeline maps Hi-C sequence data with BWA v0.7.17 (Li 2013) for mapping, then uses Samtools v1.9 (Li et al. 2009) for indexing, parsing and filtering, and Picard v3.0 (Broad Institute 2019) to tag read groups. Initial scaffolding was done with SALSA v2.3 (Ghurye et al. 2017) with the BAM files from the mapping pipeline, then Hi-C files were generated using YaHS Juicer Pre (v1.2a.2-0) (Zhou et al. 2023). Manual curation of the chromosomes was done using Juicebox v2.20.00 with the aid of juicer v1.6 and juicer_tools v1.9.9 (Durand et al. 2016) as described in their methodology. Genome quality was assessed using multiple quality metrics, including BUSCO v5.4.6 with the embryophyta_odb10 database (Berkeley et al. 2021) for gene completeness and duplication frequency, Merqury v1.3 (Rhie et al. 2020) for k-mer usage completeness, the LTR Assembly Index (LAI) v2.9.0 (Ou et al. 2018) for LTR-based quality assessment, and GASS 1.2.0 (Wang et al. 2015) for genome assembly statistics.

### Genome annotation of repeats

RepeatModeler v2.0.4 was used for *Cuscuta*-specific de novo repeat identification, and results were combined with repeats from the Dfam database (RepeatMasker v4.1.5). RepeatMasker was then employed to annotate repeats, generate repeat landscapes, and mask the genome for subsequent annotation of protein-coding genes and other analyses (Tarailo-Graovac and Chen 2009; Flynn et al. 2020). A more detailed LTR annotation was performed using LTR_FINDER (Xu and Wang 2007) and integrated into the RepeatMasker repeat annotation using RepeatCraftp (Wong and Simakov 2019) for subsequent calculation of the LAI (Ou et al. 2018). Additionally, an alternative repeat annotation was conducted using earlGrey (Baril et al. 2024).

### Genome annotation of protein-coding sequences

External transcript data for *C. campestris* (Ranjan et al. 2014; Vogel et al. 2018) and *Cuscuta australis* (Sun et al. 2018; Vogel et al. 2018) were incorporated into the annotation pipeline as evidence, with transcript data mapped using STAR v2.7.1 (Dobin et al. 2013). Protein-coding gene annotations were generated using the BRAKER pipeline (Stanke et al. 2006, 2008; Hoff et al. 2016; Hoff et al. 2019; Brůna et al. 2021) using iterative gene modeling with Augustus and NCBI BLAST (Altschul et al. 1990; Camacho et al. 2009; Li et al. 2009; Barnett et al. 2011; Dobin et al. 2013). Additional extrinsic evidence supporting the annotation process was provided by protein homology data from various flowering plant orders (Asterales, Apiales, Boraginales, Brassicales, Caryophyllales, Cornales, Ericales, Gentianales, Lamiales, and Solanales) and incorporated into the BRAKER pipeline (Supplementary File 1) (Lomsadze et al. 2005; Gotoh et al. 2014; Brůna et al. 2020; UniProt Consortium 2021). Gene model refinements and UTR annotation were performed using the PASA pipeline (Haas et al. 2003, 2008; Campbell et al. 2006). Final gene model IDs were named following Zhang et al. (2024) and annotations converted to GFF3 using AGAT (Dainat 2022). The assessment of annotation completeness was evaluated using BUSCO and the embryophyta_odb10 database (Berkeley et al. 2021). Annotation quality statistics were calculated and compared between the annotations of the *Cuscuta* species, as well as 2 *Ipomoea* species (*I. cairica* and *I. nil*) using AGAT (Dainat 2022).

### Genome annotation of small RNAs

Endogenous small RNA loci were annotated using existing small RNA-seq data from in vitro, host-free tissues (Hudzik et al. 2023) with ShortStack (version 4.0.1) (Johnson et al. 2016) run under default parameters. The results were then cross-referenced against previous *C. campestris* miRNA annotations using prior genome assemblies, manually inspected, and placed into families based on miRNA sequence similarities.

### Identification of mobile RNAs

**Fig. 2.**
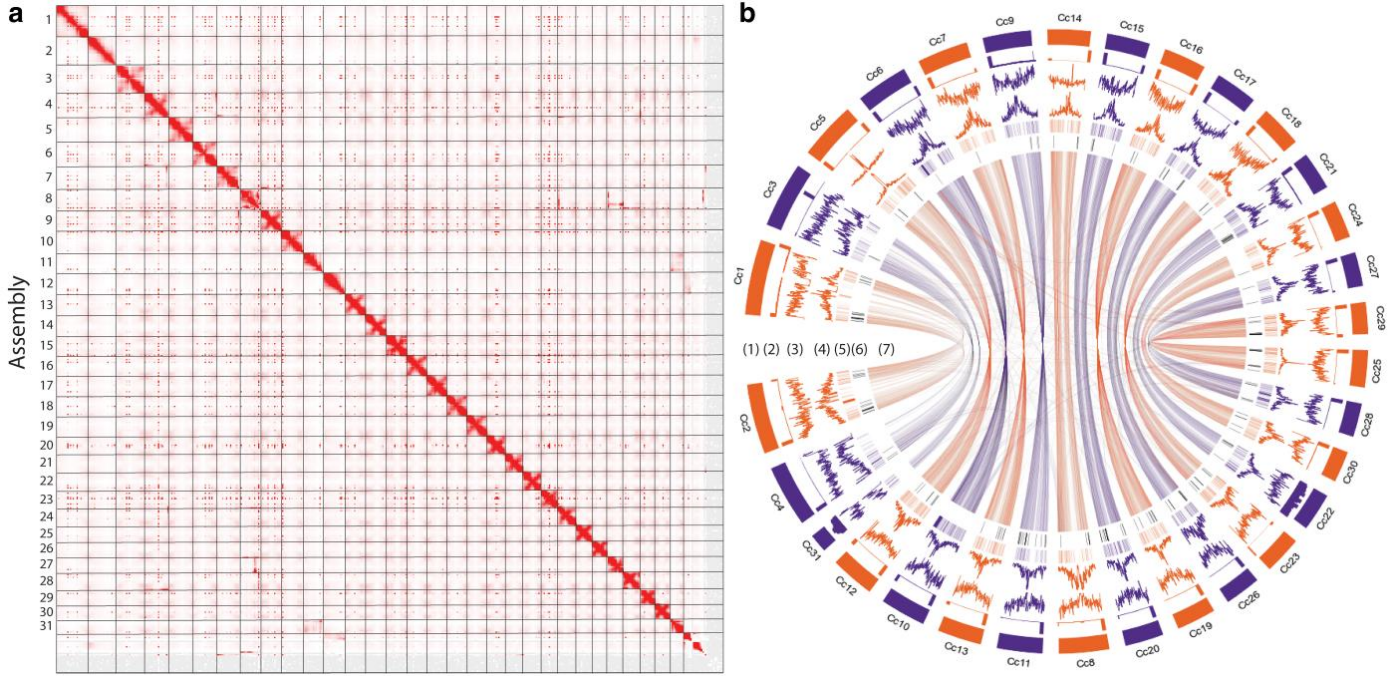
Genome assembly of *C. campestris*. a) Contact map showing 31 distinct chromosomes. b) Visualization of genome, (1) chromosomes, (2) telomere repeat sequence distribution (window size = 30,000 bp), (3) gene content distribution (window size = 10,000 bp), (4) LTR content distribution (window size = 10,000 bp), (5) distribution of mobile mRNAs, (6) distribution of miRNAs, and (7) syntenic blocks connecting the chromosomes.

*C. campestris* unigenes identified as mobile in Kim et al. (2014) were mapped to their corresponding annotated genes using BWA mapping (Li 2013). The genomic locations of genes with mobile transcripts were illustrated on the Circos plot (Fig. 2b).

### Genome structure and comparative analysis

Whole genome alignment to *I. cairica* (Jiang et al. 2022) was performed using NUCmer (Marçais et al. 2018) and visualized using dotPlotly in R (Poorten 2018). Telomeric sequence distribution was calculated with the Telomere Identification toolkit (tidk) with a 3,000 bp window size (Brown et al. 2023). Synteny analysis between chromosome sequences in *C. campestris* was conducted using MCscanX (Wang et al. 2012). Visualization of telomeric sequence distribution, gene content distribution, repeat element distribution, mobile mRNA distribution, miRNA distribution, and synteny between chromosomes was performed with pyCircos (Hideto et al. 2022).

Gene models for *C. campestris* were classified into orthogroups (OGs, objective gene families) using the GeneFamilyClassifier module of PlantTribes2 (Wafula et al. 2023), selecting best hits supported with both HMM and BLAST searches. This classification was based on the PlantTribes2 37-genome gene family scaffold created with OrthoFinder (Emms and Kelly 2015, 2018b, 2019) and contained a broad distribution of angiosperm genomes that should be optimal for classifying the *Cuscuta* lineage genes into it. In addition, gene models for Convolvulaceae [*C. campestris* (Vogel et al. 2018), *C. australis* (Sun et al. 2018), *I. cairica* (Jiang et al. 2022), *I. nil* (Hoshino et al. 2016), and *Ipomoea purpurea* (Lyons et al. 2008; Lyons and Freeling 2008; Gupta et al. 2023)], Solanaceae species [*Capsicum annuum* (Hulse-Kemp et al. 2018), *S. lycopersicum* (Fernandez-Pozo et al. 2015; Hosmani et al. 2019), and *Solanum tuberosum* (Hardigan et al. 2016)] as well as *Coffea canehora* (Denoeud et al. 2014) from Rubiaceae as an outgroup, were also classified into OGs. Gene models from Convolvulaceae, Solanaceae, and Rubiaceae species classified into the PlantTribes2 37-genomes scaffold were independently circumscribed into OGs using OrthoFinder2 (Emms and Kelly 2018b), to create a narrow gene classification of closely related lineages. CoRe OrthoGroups (CROGs) analysis was performed to evaluate the consistency of gene family size across the annotations, as described by Wafula et al. (2023).

### “Lost orthogroup” analysis

To investigate patterns of gene loss in *Cuscuta*, we examined PlantTribes2 OGs that contained at least one gene from all nonparasitic plants and none in either *C. campestris* or *C. australis*. All *Arabidopsis thaliana* reference genes in the OG were then utilized for functional analysis in DAVID (Huang et al. 2009; Sherman et al. 2022) to determine enriched functional terms associated with gene loss in *Cuscuta*.

### Phylogeny

A total of 650 single-copy OGs, contained in at least 70% of classified species from the Convolvulaceae, Solanaceae, and Rubiaceae families, were used for species tree inference. Multiple sequence alignments of OGs were estimated with the MAFFT algorithm (Katoh and Standley 2013) using the PlantTribes2 GeneFamilyAligner module, and phylogenetic trees were inferred with RAxML (Stamatakis 2014) using the PlantTribes2 GeneFamilyPhylogenyBuilder module. A coalescent species tree was inferred from single-copy genes using Astral with 1,000 replicates of bootstrap re-sampling (Zhang et al. 2018). Additionally, a concordant species tree was inferred using an alternative algorithm, Species Tree Inference from All Genes (STAG), implemented in OrthoFinder2 (Emms and Kelly 2018a, 2018b).

### Host growth and C. campestris inoculation

*A. thaliana* Col-0 host plants for *C. campestris* were grown in a short-day walk-in chamber (10 h light, 14 h dark, and 22 to 24 °C) with a light intensity of 160 to 180 µmolm^−2^s^−1^. Transgenic RUBY *Cuscuta* (*35S:RUBY*) and wildtype (WT) *Cuscuta* were grown on host beets (*Beta vulgaris*) in a long-day walk-in chamber (16 h light, 8 h dark, and 25 to 30°C) with a light intensity of 160 to 180 µmolm^−2^s^−1^. To promote host and *Cuscuta* growth, plants were grown under full-spectrum LED (Kihung LED, model: KHT-T8G003-V8) and far-red enriched lights (GE R30, model: 30711). Around a month after inoculation, 3 to 5 cm of the apical tissue of RUBY *Cuscuta* and WT *Cuscuta* were collected and re-inoculated onto flowering Arabidopsis Col-0. *Cuscuta* stems were allowed to grow and produce haustoria.

### Sectioning and microscopy

Green fluorescent was observed in RUBY *Cuscuta* and WT *Cuscuta* using eGFP filter set (450 to 490 nm excitation and 500 to 550 emission) with a widefield microscope (Leica Thunder Stereomicroscopy System). Two-week-old RUBY *Cuscuta* (*35S:RUBY*) stems and haustorial regions parasitizing Arabidopsis Col-0 were harvested and embedded in 5% agarose (Park et al. 2022). The tissues were sectioned at a thickness of 100 µm using a vibratome (Leica ARTOS 3D ultramicrotome) according to the manufacturer's protocol and imaged with widefield (Leica Thunder Stereomicroscopy System) and confocal (Leica TCP SP8 STED and MP) microscopes. To visualize RUBY fluorescence within the host stem, the sectioned tissue was excited at 514 nm, and its yellow fluorescence was emitted at 527 nm by the Yellow Flourescent Protein (YFP) filter. Following Z-stack acquisition using UV illumination, transmitted light, and a YFP fluorescence filter, the resulting image sets were subsequently merged for analysis.

## Results and discussion

### Genome assembly

We generated an initial 520 Mb, gapless assembly of PacBio HiFi reads with 385 contigs, a max contig length of 27.6 Mb, and a contig *N*_50_ of 16.2 Mb. Compared to the prior assembly of *C. campestris* by Vogel et al. (2018) (size = 477 Mb, max contig = 0.4 Mb, contig *N*_50_ = 0.05 Mb), our genome resulted in a more contiguous assembly, with some contigs representing complete chromosomes (Table 1). Our initial assembly is more similar to the *C. australis* assembly by Sun et al. (2018), which has a small number of primary contigs, large *N*_50_ and contig length (size = 273 Mb, max contig = 10.2 Mb, contig *N*_50_ = 3.6 Mb).

**Table 1.** Convolvulaceae genome assembly comparisons.

Assembly	*C. campestris*	*C. campestris*	*C. australis*	*I. cairica*	*I. nil*
Study	This study	Vogel et al. Nat Commun (2018)	Sun et al. Nat Commun (2018)	Jiang et al. G3 (2022)	Hoshino et al. Nat Commun (2016)
Number of chromosome corresponding sequences (*N*)	31	—	—	15	15
Prior estimated number of chromosome sequences (*N*)	30	30	15	15	15
Number of scaffolds	372	6907	103	75	3416
Number of contigs	385	27,832	249	78	3865
Est. genome size (Mb)	557	557	272.57	730	750
Scaffold sequence (Mb)	520.045	476.795	264.83	733.045	734.803
Contig sequence (Mb)	520.045	450.73	—	733.043	734.591
Scaffold *N*_50_ (Mb)	16.184	1.385	5.95	45.7	2.88
Contig *N*_50_ (Mb)	16.184	0.049	3.626	43.8	1.873
Max scaffold (Mb)	27.583	5.342	10.193	65.792	14.45
Max contig (Mb)	27.583	0.401	10.193	65.792	9.127
Assembly BUSCO complete rate (%)					
Complete	89.2	88.9	87.7	98.1	98.0
Complete and single copy	12.0	20.3	85.7	93.3	93.0
Complete and duplicated	77.2	68.6	2.0	4.8	5.0
Fragmented	0.5	0.9	1.5	0.6	0.7
Missing	10.3	10.2	10.8	1.3	1.4
LAI (whole genome)	10.57	3.07	11.88	5.47	5.21

*C. campestris* genome assembly statistics and comparison with prior assembly, a second *Cuscuta* species and 2 nonparasitic, closely related species in Convolvulaceae. BUSCO scores calculated using genome mode BUSCO.

The genome-based BUSCO profile showed 89.2% completeness, comprising 77.2% complete and duplicated genes and 12.0% single-copy genes. This high level of gene duplication is similar to the *C. campestris* assembly of Vogel et al. (2018) (C: 88.9% [D: 68.6%, S: 20.3%]), but it differed from the *C. australis* assembly by Sun et al. (2018), which predominantly contains single copy genes (C: 87.7% [D: 2.0%, S: 85.7%]). These findings are consistent with a genome duplication in *C. campestris*, as previously reported (Vogel et al. 2018).

All *Cuscuta* genome assemblies have an increased number of missing genes in their BUSCO profiles (*C. campestris* Cerda = 10.3%, *C. campestris* Vogel = 10.2%, *C. australis* Sun = 10.8%), similar to the BUSCO profiles of other parasitic and mycoheterotrophic plants (Yuan et al. 2018; Xu et al. 2021; Timilsena et al. 2023). As a relevant comparison to closely related nonparasitic species, both *I. cairica* and *I. nil* have a complete BUSCO score of around 98%, with most genes detected as complete and in single copy.

We then generated a chromosome-level assembly by incorporating chromosome conformation capture sequencing (Illumina Hi-C) data, resulting in a 520 Mb assembly with 372 scaffolds (scaffold *N*_50_ = 16.2 Mb, max scaffold length = 27.6 Mb), of which the 31 largest scaffolds represent pseudochromosomes (hereafter referred to as chromosomes) (Fig. 2a). Our 520 Mb assembly was close to the genome size estimated by flow cytometry of 557 Mb for *C. campestris*, suggesting a higher level of sequence completeness and representation than the prior genome (Table 1) (Vogel et al. 2018). Published chromosome counts for *C. campestris* are 1C = 28 or 30 (Ibiapino et al. 2022); however, our assembly identified a total chromosome number of 1C = 31, with a length of 505 Mb. Heterozygosity was estimated to be 0.352%, which is consistent with its inbred life history and subsequent self-fertilizations in the laboratory (Supplementary Fig. 1).

We aligned our *C. campestris* assembly to the chromosome-level assembly of *I. cairica* to determine chromosome structure conservation and homology. Most chromosomes in *I. cairica* correspond to 2 full chromosomes, suggesting genome duplication in *C. campestris* and for the most part, chromosome structure is conserved (Supplementary Fig. 2).

### Genome annotation

We generated a de novo annotation for the *C. campestris* genome, resulting in 47,199 protein-coding gene models across the 31 chromosomes, 1,374 more than the prior genome annotation (Table 2). The statistics for the annotation, including mean exon number, mean exon length, and mean CDS length (among others), were comparable to high-quality annotations of related species of *Cuscuta* and nonparasitic plants (Table 2).

**Table 2.** Convolvulaceae annotation statistics comparison.

	*C. campestris*	*C. campestris*	*C. australis*	*I. cairica*	*I. nil*
Gene prediction	(This study)	(Vogel et al. 2018)	(Sun et al. 2018)	(Jiang et al. 2022)	(Hoshino et al. 2016)
Gene number	47,199	45,825	18,157	38,115	35,151
Average exon number	5	5.7	5.5	4.68	4.9
Average exon length (bp)	330	325	226	236	273
Total exon length (bp)	77,539,792	84,984,072	22,471,923	42,156,936	47,058,378
Average CDS length (bp)	1,228	1,253	1,237	1,106	1,338
Annotation BUSCO assessment (%)					
Complete	90.1	89.4	87.3	98.5	99.3
Complete and single copy	10.3	17.1	83	93.3	94.2
Complete and duplicated	78.6	66.2	1.9	5.2	5.1
Fragmented	1.2	6.1	2.4	0.9	0.1
Missing	9.9	10.6	12.7	0.6	0.6

*C. campestris* genome annotation statistics and comparison with prior assembly, a second *Cuscuta* species and 2 nonparasitic, closely related species in Convolvulaceae.

Annotation-based BUSCO statistics were very close to the genome-based BUSCO statistics, suggesting a high level of annotation completeness (Table 2). The annotation-based BUSCO scores showed a profile dominated by complete and duplicated genes, followed by complete and single-copy genes (C: 90.1 [S: 10.3, D: 78.6] F: 1.2, M: 9.9), just like the genome-based BUSCO scores (Tables 1 and 2). The annotation-based BUSCO statistics are comparable to or higher than other holoparasites (Frailey et al. 2018; Guo et al. 2023) or mycoheterotrophs (Yuan et al. 2018; Xu et al. 2021; Timilsena et al. 2023), which consistently show a large number of missing genes; all *Cuscuta* species analyzed in this study were missing approximately 10% of otherwise conserved BUSCO genes.

To facilitate accurate cross-identification of genes for analytical or experimental purposes, we created an M:N table (Supplementary File 2) comparing the number of genes in our annotation (M) and the Vogel et al (2018) prior annotation (N), using BLAST-based ortholog classification with OrthoFinder2 (Emms and Kelly 2015, 2018b). For this table, 45,754 genes of 47,199 total were classified into OGs of at least 2 genes, approximating 96.9% of our annotation, similar to the 44,024 (96.1%) classified from the prior annotation. The number of OGs populated by genes from this annotation and Vogel et al.’s (2018) annotation were 22,013 (57.4%) and 21,650 (56.4%), respectively, which was higher than the rest of the *Cuscuta* species (Supplementary File 3). There was a similarly high assignment of genes into OGs for our annotation (41,006, 89.6%) and the Vogel et al. (2018) assembly 40,976 (93.1%). However, fewer genes were assigned to the same OGs in both genomes (24,404, or 53.3% for our annotation and 64.5% for the Vogel et al. (2018) annotation).

There are substantial differences in the 2 annotations of *C. campestris*, despite having very similar BUSCO scores and a 94.4% sequence identity between the 2 sequenced genomes. It is known that different methodologies in the annotation process can lead to drastically different results in the gene models and to inconsistent gene prediction (Chen et al. 2020). Therefore, a close examination of gene modeling and filtration methods should be undertaken in future research.

### miRNA annotation

miRNAs were annotated based on previously described *C. campestris* small RNA-seq data (Hudzik et al. 2023). The miRNAs of *C. campestris* are of particular interest because many of them accumulate only in haustorial tissue and, uniquely to our knowledge, have primary transcripts made by RNA polymerase III (Hudzik et al. 2023). Several of these interface-induced miRNAs function to target host mRNAs (Shahid et al. 2018; Johnson et al. 2019). A total of 146 interface-induced miRNA loci, representing 96 distinct families of mature miRNAs, were annotated (Supplementary File 4). Additionally, several canonical miRNA loci (68 loci representing 22 distinct families) were annotated, including several families widely conserved in flowering plants, as well as a few seemingly species-specific families. Canonical miRNA loci are not interface-induced and have no evidence of Pol III transcription. The miRNA annotations are generally in agreement with prior miRNA annotations performed on earlier genome assemblies (Subhankar et al. 2021; Zangishei et al. 2022; Hudzik et al. 2023).

### Identification of mobile mRNAs

Mobile mRNAs are thought to function as systemic signals that may contribute to coordinating plant development and responses to stress (Kehr et al. 2022). The exact number of mobile transcripts and the precise mechanisms that determine their mobility and function remain active topics of research. Nevertheless, hundreds to thousands of genes produce mobile transcripts, and that mobility is influenced by factors such as the plant species in question, the growth stage of the plant, and whether it is under stress. *Cuscuta* is especially interesting with respect to RNA mobility because of the potential functional significance of translocated RNAs in enhancing the successful colonization of the host (Shahid et al. 2018) and because the genetic differences between the parasite and its host create an ideal system for tracking cross-species movement of RNAs (Kim and Westwood 2015). To consider the genomic context of mobile transcripts, we mapped previously identified *C. campestris* unigenes that were mobile into Arabidopsis (Kim et al. 2014) to a total of 3,238 genes in the new *C. campestris* genome (Fig. 2b and Supplementary File 5). The results suggest that genes with mobile transcripts generally correspond to the overall density of protein-coding genes. However, notable clusters of mobile transcript-encoding genes, for example, on chromosomes 6, 10, and 28, suggest that certain regions of the genome may be enriched in the production of interspecies mobile transcripts.

### Genome structure and repeat analysis

Synteny analysis within *C. campestris* shows that each chromosome has one mostly full-length, structurally conserved homeolog, except for the homeolog of chromosome 3, which appears to have been split into chromosomes 4 and 31 (Fig. 2b and Supplementary Fig. 2). We found no evidence in the Hi-C data for chromosomes 4 and 31 to be joined together (Supplementary Fig. 3a). We identified HiFi reads mapping to either end of chr4 and chr31, where the highest number of reads was only 199 at the end of the 2 chromosomal sequences, and not enough to scaffold them together. We also looked at syntenic blocks shared between chr4 and chr31 and their homolog chr3 and found some syntenic blocks at the end of chr4 and chr31 that are not shared with chr3 (Supplementary Fig. 3b). This could point to a previous segmental duplication as the cause of the chromosomal splitting. Sequence identity between the homeologous chromosomes was, on average, 93.2%, with individual homoeologous pairs (other than the more divergent chromosome pair #3 and #4, #21) ranging from 91.8% to 93.5% identical (Supplementary Fig. 4). This, together with the high retention of complete and duplicated genes from BUSCO analysis, supports the conclusion that the genome duplication was quite recent, in concert with the molecular clock-based estimate that the genome duplication occurred approximately 5 MYA (Vogel et al. 2018).

**Fig. 3.**
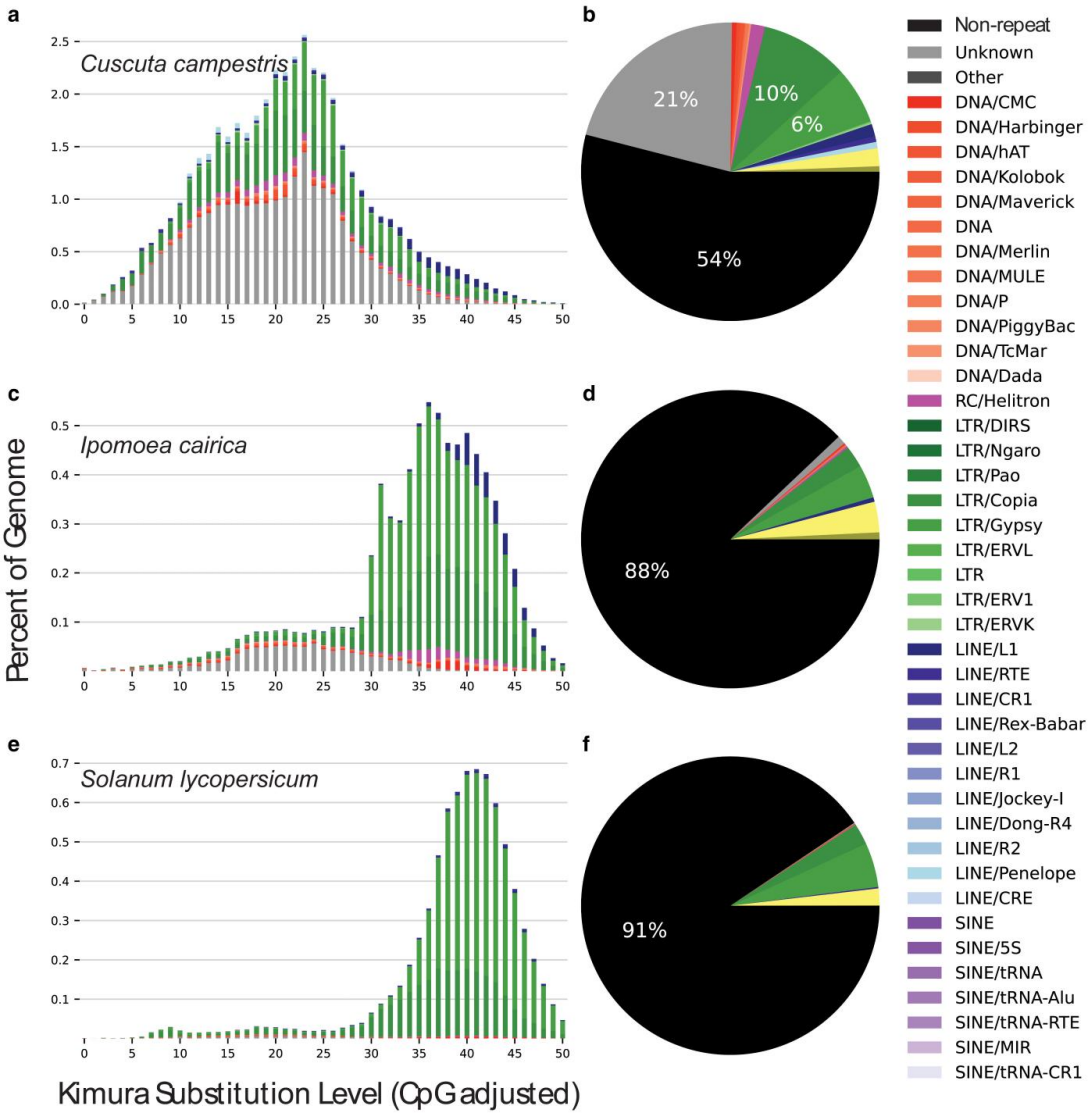
Repeat analysis and comparison between the parasitic plant *C. campestris* (this study, Convolvulaceae), the closely related, nonparasitic species *I. cairica* (Jiang et al. 2022; Convolvulaceae) and a more distantly related nonparasitic species *S. lycopersicum* (Fernandez-Pozo et al. 2015; Hosmani et al. 2019; Solanaceae). a), c), and e), showing the repeat landscape for the 3 species: *X* axis showing the Kimura substitution level (KSL) for among-copy divergence, and *Y* axis showing the percent of the genome occupied by each repeat at each KSL. b), d), and f), showing the percentage of genome occupied by any given type of repeat. Black = nonrepeat regions.

We analyzed and compared repeat landscapes across Convolvulaceae and tomato to determine changes in repeat content. Repeat landscapes for *I. cairica* and *S. lycopersicum* showed a pattern of repeats similar to those of most plants that lack active repeat elements (Fig. 3c to f). Repeats primarily encompassed the transposable elements Gypsy and Copia, with approximately 10% of the genome composed of repetitive elements. In contrast, the *C. campestris* genome was 46% repetitive elements, of which 15.7% were LTR Gypsy and Copia, and 21.2% were from unknown sources (Fig. 3a and b and Supplementary File 6). The repeat landscape also suggests that the *Cuscuta* repeat expansion events are more recent than those in nonparasitic species, based on the Kimura Substitution Level of among-copy divergences (Fig. 3a, c, and e). *C. australis* has a repeat distribution pattern similar to that of *C. campestris*, indicating that the expansion of repeats had begun before the speciation of these 2 *Cuscuta* species (Supplementary Fig. 5).

We analyzed telomeric sequence distributions across the assembly and found that 27 of 31 chromosome sequences have telomeres at both ends, providing further evidence for a high-quality assembly. Three of the remaining 4 (#3, #4, and #21) have a telomere peak at one end. Additionally, chromosome 3 shows a telomeric sequence peak within the chromosome sequence but does not appear to be shared with the homeologous chromosomes, since chromosome 4 has a single peak at one end, while chromosome 31 has a low but even distribution of telomeric sequences. Chromosome 21 also has an even distribution of telomeric sequences, contrasting with its homeologous, chromosome 22, which has a single telomeric peak at one end (Fig. 2b). The uneven distribution of telomeric sequences in chromosomes 31 and 4 can be explained by the splitting of chromosome 3 into 2 separate chromosomes, which also elucidates the unexpected extra 31st chromosome.

Gene content distribution was generally even across all chromosomes, with a slight dip in concentration closer to where centromeres are expected. LTR distribution was similarly even throughout the chromosomes, but opposite to that of gene content, showing peaks in areas where centromeres are expected (Fig. 2b). The averaged whole-genome LAI score was 10.57, the second largest value after *C. australis* (11.88), which qualifies our genome as a “reference” quality assembly (Table 2) (Ou et al. 2018).

### CROGs analysis: enrichment and loss

**Fig. 4.**
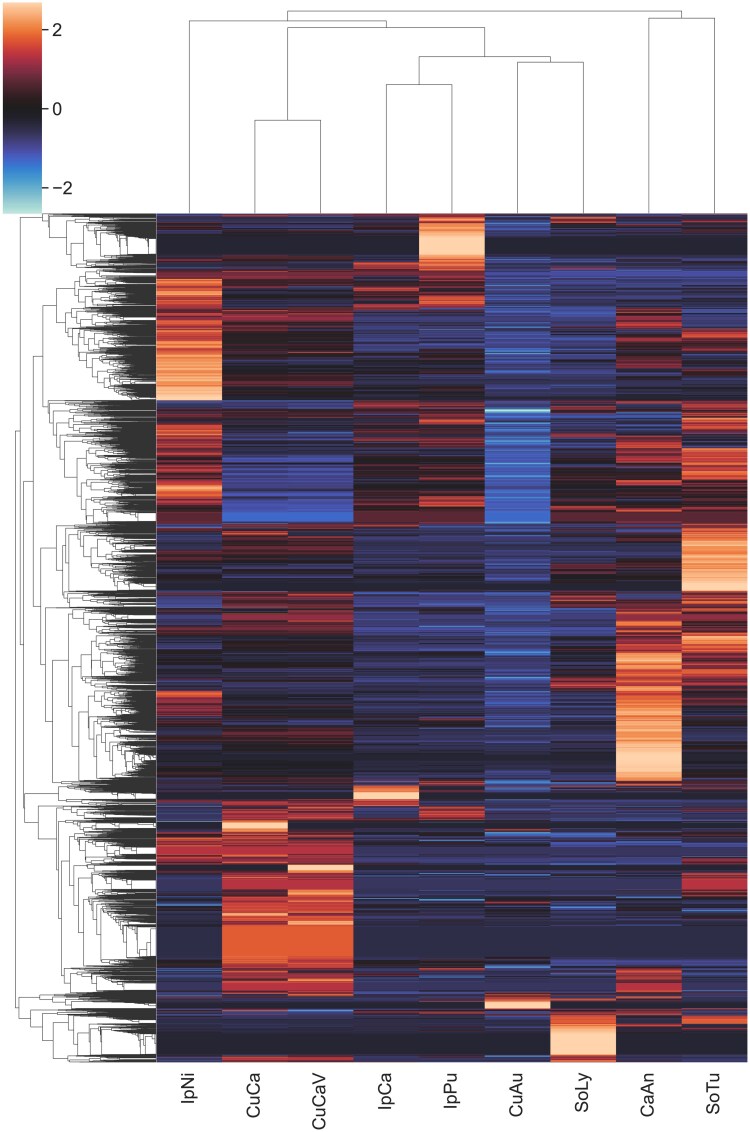
CoRe OrthoGroups (CROGs) analysis (Wafula et al. 2023) showing clustering of species by *Z*-score based on the number of annotated genes within given OGs. Redder and yellower OGs are expanded in gene number relative to other taxa, while Bluer OGs are reduced in gene number relative to other taxa. CuAu = *C. australis* (Sun et al. 2018), CuCa = *C. campestris* (this study), CuCaV = *C. campestris* (Vogel et al. 2018), IpNi = *I. nil* (Hoshino et al. 2016), IpCa = *I. cairica* (Jiang et al. 2022), IpPu = *I. purpurea* (Lyons et al. 2008; Lyons and Freeling 2008; Gupta et al. 2023), CaAn = *C. annuum* (Hulse-Kemp et al. 2018), SoLy = *S. lycopersicum* (Fernandez-Pozo et al. 2015; Hosmani et al. 2019), and SoTu = *S. tuberosum* (Hardigan et al. 2016).

We used CROGs analysis to compare annotations and find biological or methodological patterns of OG reduction or expansion across Convolvulaceae and Solanaceae (Wafula et al. 2023; Zhang et al. 2024). PlantTribes2 classified the genes into OGs on a broader taxonomic scale using the 37-genome scaffold. For this second classification, 40,699 genes (86.4%) in our assembly were classified into 8,689 OGs (73.6% of total OGs), similar to 40,330 (88.0%) classified genes of the prior assembly. Overall, CROGs analysis clustered species with similar gene contents together, suggesting similarity in genome annotation (Fig. 4). *C. campestris* annotations clustered together, showing similarity in gene content throughout the OGs, and a higher, approximately double, overall number of gene models when compared to those of *C. australis*. Among *Cuscuta* genomes, regions with higher and lower CROG gene counts (higher and lower *Z*-score values, respectively) appeared to be conserved, suggesting genus-wide expansion and reduction of gene families.

**Fig. 5.**
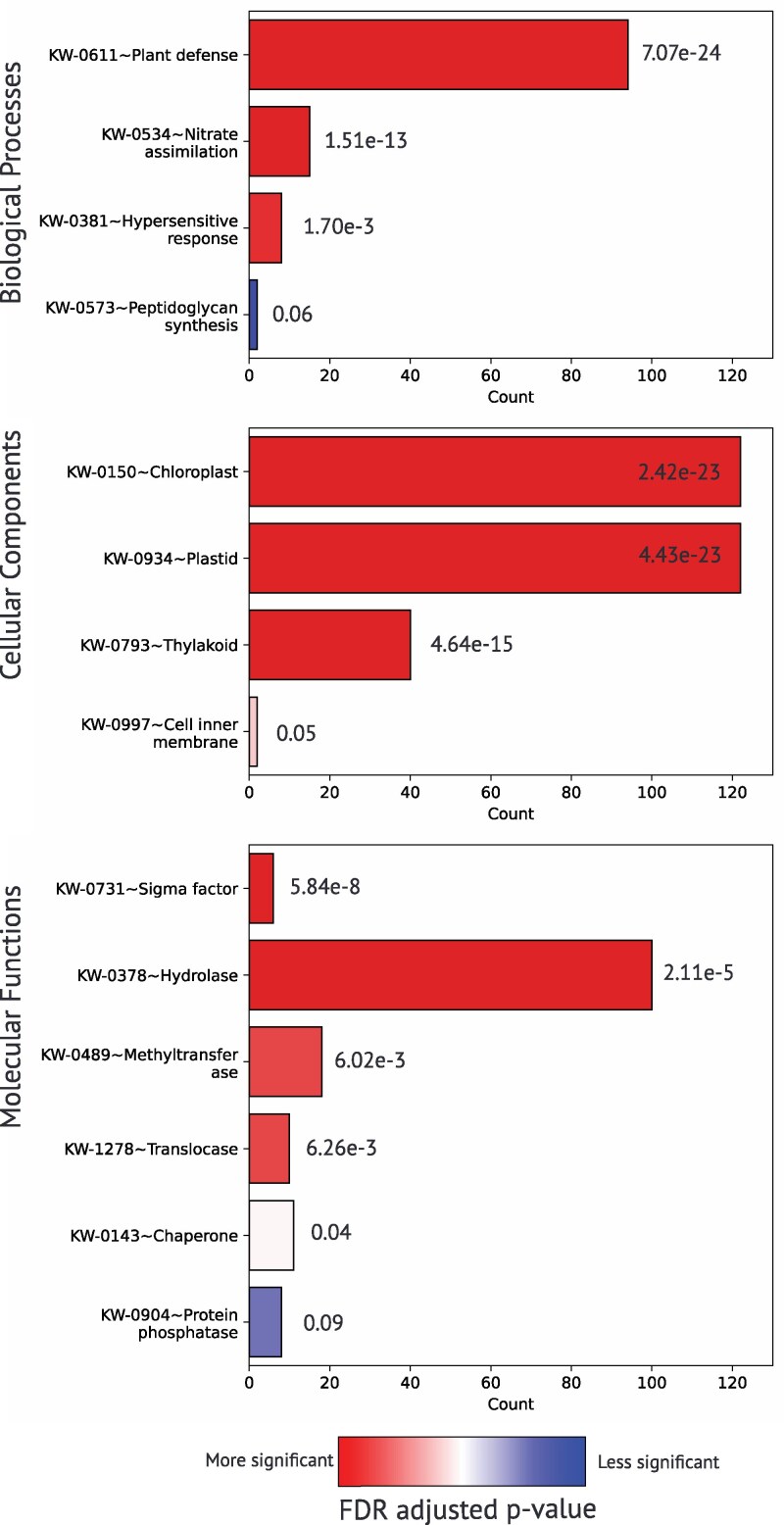
Gene enrichment analysis of “lost” genes in *Cuscuta: Molecular Functions, Cellular Components, Biological Processes. X* axis shows the count of genes in each biological term; *Y* axis shows the primary biological terms. Color of the graphs represents the FDR-adjusted *P*-value for each term.

We estimated gene loss in the genus *Cuscuta* by counting the number of genes from nonparasitic plants in OGs where *Cuscuta* species had zero genes present. A total of 694 OGs contained zero *Cuscuta* genes and at least one gene (mean ∼1.97 genes) in related nonparasitic species, meaning that the genus *Cuscuta* has lost approximately 1,367 genes from these missing OGs. We used the Arabidopsis homologs of genes from these OGs (862 *A. thaliana* genes) for an enrichment analysis of “missing” genes in *Cuscuta* to find evidence of functional loss in the genome (Fig. 5). The enrichment analysis of lost genes highlighted ontological patterns seen in previous research, primarily relating to photosynthesis and root-based nutrient assimilation (Vogel et al. 2018). Cellular components heavily highlighted gene losses related to chloroplast and cellular structures, while biological processes and molecular functions were focused more on gene loss related to root-based nutrient assimilation and plant defense in the form of hydrolases and translocases, to name a few.

We calculated gene expansion in *C. campestris* relative to *C australis* in 2 ways. First, we identified the gene families in *C. campestris* with more than twice the number of genes in *C. australis*. Our assembly and annotation identified 1,213 OGs with greater than 2× expanded gene family size, accounting for an additional 10,676 genes in *C. campestris*. We then calculated the number of additional genes by counting the number of syntelogous genes from the synteny analysis and subtracting that from the total number of gene models, giving a second estimate of approximately 17,701 additional genes in *C campestris*.

### Species phylogeny

**Fig. 6.**
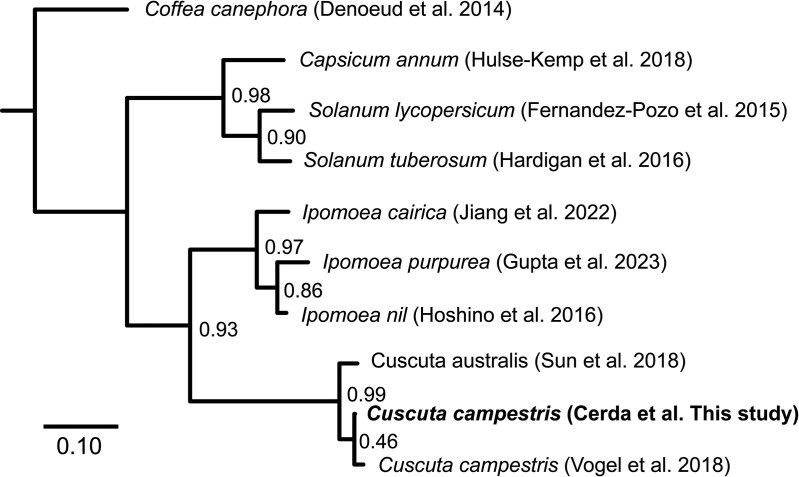
Species tree generated by OrthoFinder's STAG algorithm. Bar shows inferred substitution rate per site. Branches show STAG support value, and branches with no values have a support of 1.

We estimated species phylogenies using 2 orthology-based methods: A coalescent species tree from conserved gene trees with PlantTribes2 (Wafula et al. 2023), and another using OrthoFinder2's STAG algorithm (Emms and Kelly 2018a, 2018b). The coalescent species tree was based on 650 gene trees with at least 70% of species present in each gene tree. Both approaches estimated the same species phylogeny, with the 2 *C. campestris* genomes pairing together closely as expected, considering their near identity, along with *C. australis*, which was sister to the three *Ipomoea* specie, then Solanaceae, and finally *Coffea* (Fig. 6 and Supplementary Fig. 6).

### *C. campestris* as a model system for understanding mobile molecules

**Fig. 7.**
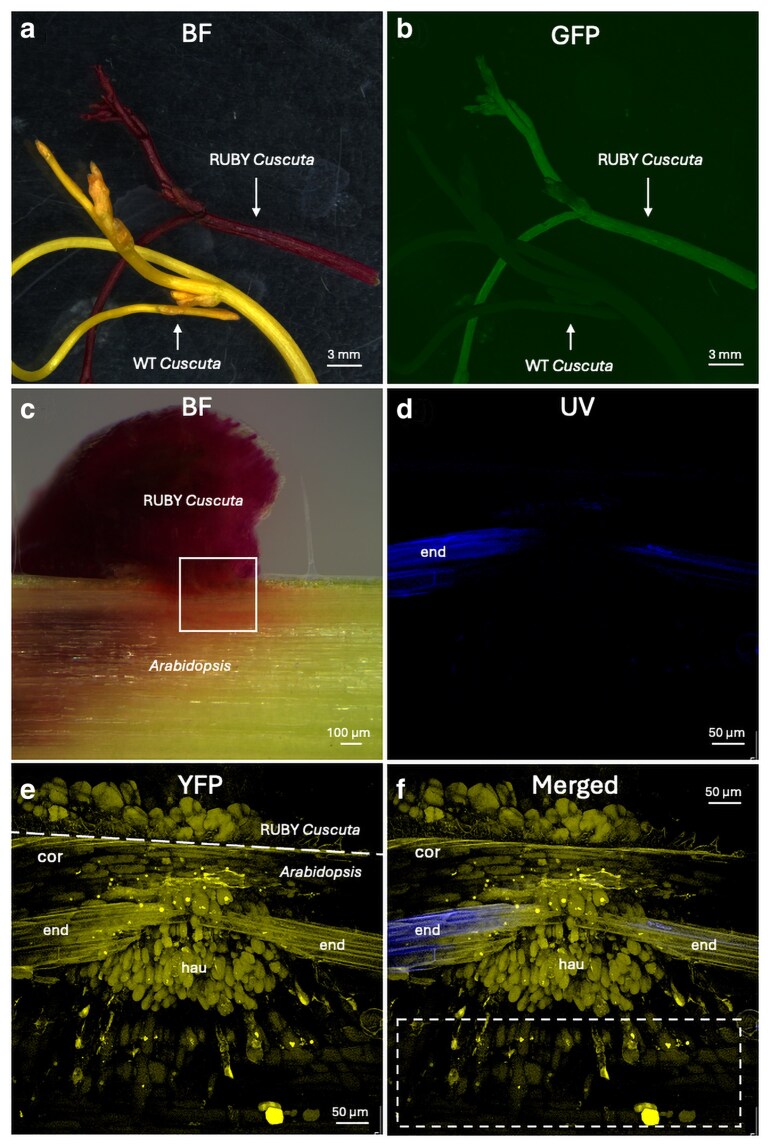
Fluorescence of RUBY observed in *C. campestris* and within the host stem. Apical tissues of RUBY *Cuscuta* (*35S:RUBY*) and WT *Cuscuta* were harvested and observed with the bright-field light a) and eGFP filter b) using a widefield microscope. Vibratome-sectioned *Cuscuta* haustoria were imaged under the widefield microscope c). Zoomed-in image of the white box in (c) was analyzed using a confocal microscope with UV d) and YFP e) filters. The obtained images from UV and YFP filters were merged to produce f). The white dashed box indicates betalain accumulations inside the host stem. Abbreviations: cor, *Arabidopsis* cortex; end, *Arabidopsis* endodermis; hau, *Cuscuta* haustoria.

*C. campestris* is the only stem-parasitic plant known to be stably transformed with the RUBY reporter system (Adhikari et al. 2024). Notably, host stems parasitized by the transgenic RUBY *Cuscuta* exhibited accumulated betalain in the near-vascular-bundle region of the host, indicating that RUBY could be a potential marker for tracking mobile molecules. RUBY can be used as a visible reporter, as it produces betalain from tyrosine (He et al. 2020), but a component of betalain, betaxanthins, has been reported to exhibit fluorescence under blue light (excitation: 450 to 490 nm) (Guerrero-Rubio et al. 2020). In this study, we investigated whether RUBY *Cuscuta* was fluorescent and whether fluorescence betalain can be detected in the host stem. Using the eGFP filter set, no fluorescence was observed in WT *Cuscuta*. However, RUBY *Cuscuta* exhibited clear green fluorescence (Fig. 7a and b). These findings suggested that RUBY can function as both visible and fluorescent markers for tracking mobile molecules between *Cuscuta* and host plants. To investigate whether betalain accumulated in the host stem also exhibited fluorescence, we collected *Cuscuta* haustoria tissues growing on Arabidopsis and observed fluorescence using a confocal microscope. Sectioned haustorium tissue (Fig. 7c) was imaged using Z-stacking under UV light (Supplementary Fig. 7), a YFP filter set (Supplementary Fig. 8), and transmitted light (Supplementary Fig. 9), and all sectioned images were then merged (Supplementary Fig. 10). The UV light detected autofluorescence from Arabidopsis endodermis (Fig. 7d and Supplementary Fig. 7). The YFP filter detected fluorescent betalain in RUBY *Cuscuta* and inside the host stem, particularly in the cortex, endodermis, and *Cuscuta* haustorium (Fig. 7e and Supplementary Fig. 8). Images from the transmitted light clearly outlined the position of *Cuscuta* searching hyphae (Supplementary Fig. 9). Interestingly, the yellow fluorescence of betalain was observed in the host vascular bundle region (white dashed box in Fig. 7f and red arrows in Supplementary Fig. 8l to t), indicating that, similar to how Adhikari et al. (2024) visually observed betalain accumulation inside the host stem, the accumulation of betalain can also be tracked using highly sensitive fluorescence microscopy (Fig. 7d to f). These findings further support the utility of *C. campestris* as an excellent model for both the discovery and the tracking of mobile molecules and expand the technical toolkit that is available to exploit the *C. campestris* genome to understand interspecies molecular movements.

## Conclusion

Here, we present a high-quality assembly and annotation for *C. campestris* and the first chromosome-level assembly of any *Cuscuta* species. Our genome was assembled into 31 clearly defined chromosomes, showcasing a recent genome duplication in concordance with previous studies. *C. campestris* already serves as a model organism for the study of plant parasitism, its evolution, development, symbiosis, and population dynamics; hence, this *C. campestris* genome will become a valuable resource as a genomic reference. A reference genome for *C. campestris* will also facilitate a wide range of studies in comparative genomics, gene expression, HGT, mobile RNA, and other molecules to study intraspecies interactions, molecular markers for high resolution species and genotype identification, herbicide development, and genetic transformation, and host-induced gene silencing, all of which will contribute to the better understanding of parasitic plants and their management in environmental and agricultural settings.

## Data Availability

Seeds are available upon request. The genome assembly, long-read HiFi sequencing data and Illumina Hi-C sequencing data have been submitted to NCBI under the Bioproject PRJNA1194178. Detailed step-by-step command-line instructions for the genome assembly are provided in Supplementary File 7. The genome assembly and annotations, as well as the RNA expression data used in this project, are also available at https://plantsmallrnagenes.science.psu.edu/Studies/Cerda. This website also includes a genome browser and BLAST server. Supplemental Material included on figshare: https://doi.org/10.25387/g3.29552831.
